# RETRACTED ARTICLE: Real-time monitoring system for early stroke detection based on fog computing and enhanced deep learning techniques

**DOI:** 10.1038/s41598-025-28513-5

**Published:** 2025-12-27

**Authors:** Alaa M. Mohamed, Hanan M. Amer, Asmaa H. Rabie, Ahmed I. Saleh, Mohy-Eldin A. Abo-Elsoud

**Affiliations:** 1 Electronics and Communications Department, Faculty of Engineering, Mansoura University, Mansoura, Egypt; 2 Delta Higher Institute for Engineering and Technology, Mansoura, Egypt; 3 Computer and Control Systems Engineering Department, Faculty of Engineering, Mansoura University, Mansoura, Egypt; 4 Computer Science Department, Arab East Colleges, Riyadh, Saudi Arabia

**Keywords:** Computational biology and bioinformatics, Diseases, Engineering, Health care, Mathematics and computing, Neurology

Retraction of: *Scientific Reports*10.1038/s41598-025-28513-5, published online 29 June 2026

The Editors have retracted this Article.

After publication, concerns were raised regarding the provenance and reliability of the public datasets used in the study. The dataset “face images of acute stroke and non-acute stroke” can no longer be verified through its original source. The dataset “facial droop and facial paralysis image” [1] appears to contain identifiable facial images; however, information regarding ethical oversight of data collection or an associated data governance framework could not be confirmed, nor is it clear that clinical diagnoses were validated. Since these datasets were used for the training and validation of the proposed model, the Editors no longer have confidence in the contents of the Article. Since the consent procedures for inclusion of identifiable information in the dataset are unclear, the content of this Article has been removed to protect the privacy of the individuals.

Hanan M. Amer, Asmaa H. Rabie, and Mohy-Eldin A. Abo-Elsoud disagree with the retraction. Alaa M. Mohamed and Ahmed I. Saleh did not explicitly state whether or not they agree with this retraction.

## References

[1] Mehta, K. (2025). Facial Droop and Facial Paralysis Image. Kaggle. Retrieved 11.05.2026 from https://www.kaggle.com/datasets/kaitavmehta/facial-droop-and-facial-paralysis-image

